# The Evils of Marriage and Pregnancy in Women Who Do Not Possess Sound Pelvic Organs

**Published:** 1896-12

**Authors:** A. E. Aust Lawrence

**Affiliations:** Professor of Midwifery and Gynæcology, University College, Bristol, and Physician-Accoucheur, Bristol General Hospital


### The Bristol Medico-Chirurgical Journal.

DECEMBER, 1896.

### THE EVILS OF MARRIAGE AND PREGNANCY IN WOMEN WHO DO NOT POSSESS SOUND PELVIC ORGANS.

The Presidential Address, delivered on October 14th, 1896, at the opening of the 23rd Session of the Bristol Medico-Chirurgical Society.

BY

A. E. Aust Lawrence, M.D., C.M. Aberd.,

*Professor of Midwifery and Gynæcology, University College, Bristol, and Physician-Accoucheur, Bristol General Hospital.*

In the first place I wish to thank you for electing me your President for the ensuing year. I regard this as a double honour, being not only a compliment to myself personally, but also to the Hospital on the staff of which I have been for nearly twenty-two years.

In selecting a subject on which to address you to-night, I have chosen one which I trust will be of general interest and at the same time enable me to bring before you some of my own special work. I wish briefly to consider the evils of marriage and pregnancy in women who do not possess sound pelvic organs.

In the first place we have to consider the effect of marriage only, and secondly that of pregnancy occurring in these women.

This is the day of preventive medicine, and although I do not expect for one moment that medical men will ever have the power to veto the marriage of people, yet I am sure it is our duty to instruct the mothers of girls that there are much greater evils for them than remaining "old maids." If the family doctor would only warn these unsound people against marriage there would be far less misery in the world than there is. The fact is, there are numbers of women who marry and yet are absolutely unable to lead a married life and can never become pregnant, and there are also a large number to whom pregnancy means a very great risk indeed.

The chief point I wish to emphasise is, that no woman whose menstrual functions are not performed normally should marry unless she has had a doctor's consent and it has been considered that neither marriage nor pregnancy will intensify her trouble. Marriage has been a very common prescription for neurotic and hysterical women, without the slightest attempt being made to ascertain whether there is any real pelvic trouble to cause the symptoms complained of.

I have notes of a large number of cases where marriage has been the cause of intense suffering both bodily and mental, and in all these cases a properly conducted examination would have revealed the absolute necessity of forbidding marriage. We all have seen cases where a life-long misery for two people might have been avoided if only a doctor had been consulted on this very important step in life. Frequently the family doctor is asked whether a certain woman might marry, and he interrogates every organ except those which have to bear the brunt of married life, and he passes women as sound who are sound practically for all purposes save that of marriage. I have had some amusing cases of elderly females who have passed the climacteric asking me if they might marry. I have generally said "yes" provided that, as in life insurance policies, the "age is admitted."

What has struck me as so peculiar is, that women who suspect that they are wrong in the pelvic region will not consult a doctor as to whether they may marry or not, and they resent any opinion given that they are not as other women.

Only very recently in the Hospital I told a woman who had no vagina, uterus or appendages that she was not a fit or proper person to marry, and yet in spite of this opinion she did marry.

The first set of cases I wish to allude to is a very common one and productive of the greatest misery. I allude to women marrying who have had such an attack of pelvic inflammation as to leave the pelvic organs matted together and fixed. I might mention here that measles and scarlet fever in adult women are common causes of this form of pelvic inflammation. I have been consulted frequently by women on account of painful menstruation which has only been set up since an attack of measles or scarlet fever, and I have invariably found in these people evidence of pelvic disorder, most frequently fixation of one or other uterine appendage. If such a woman marries she lights up again the old mischief. She has intense dyspareunia: this term, coined by Dr. Robert Barnes, signifies painful performance of the marriage act, and as he says, "however disagreeable the topic may be, it is impossible to escape reference to a function so important." If she becomes pregnant she frequently aborts. I have seen a large number of these women, some *before* marriage, have warned them (without any avail) not to marry, and have seen them *after* marriage when they were made very much worse. I have also seen these cases for the first time after marriage, and from the previous history have been convinced that there was mischief prior to marriage which had been made worse since.

At this stage let me protest against the man being always made the scapegoat for these inflammatory attacks. I am well aware that a latent gonorrhœa or gleet does often set up a train of symptoms like those described, and I have seen cases where, from the clinical history prior to marriage, I am sure the woman was healthy and that the symptoms were due to infection from the husband; and here I must ask those gentlemen who look after the men to bear in mind that there are certain men who ought not to marry, as they would be a distinct source of danger to their prospective wives.

In the present day it is the fashion to regard pelvic inflammatory attacks occurring in young married women to be the result of infection from the husband. My own cases do not bear out this to anything like the extent that is commonly supposed. It is difficult to get the history of the young wife's ailments prior to marriage, and her mother will resent any idea that she, the daughter, was not perfect in every respect, and in proportion as she knows she was not, so she blames the non-erring husband. Of course he is indirectly the cause of the old mischief lighting up, but he is blameless in the matter. My experience is, that it is a common practice for mothers to make light of pelvic disorders in their daughters, and if mischief happens after marriage to throw the whole blame on the husband. Mothers ought to take a common-sense view of this question, and if their daughters' menstrual history is not normal then the family doctor should be consulted. An examination of the pelvic organs sufficient for all purposes that I now advocate can be made through the rectum and abdominal wall, and I consider it ought to be made in all doubtful cases. I am convinced that marriage would not be thought of in a large number of the cases if the doctor consulted would just let the mother and the daughter know of all the evil effects likely to ensue from marriage. Of course there are many pelvic states which produce painful menstruation, some of which would not interfere with marriage, but without a pelvic examination it is impossible to differentiate them.

I now take another set of cases where the effect of marriage is not as a rule to intensify the condition that is abnormal, but sterility or repeated abortions are common and parturition is increased in its danger.

I allude to cases of fibro-myoma of the uterus. In a certain proportion of these cases there are no symptoms whatever to lead the woman to suppose that anything is wrong, and it is only after some years of married life that she consults a doctor and finds that she is not likely to become pregnant. In these cases there is no dyspareunia, and except for the sterility the conditions of married life are normal.

I have no remedy to suggest for these cases; although if a man married a woman so affected with the idea of having an heir to his title and estate, it certainly would be highly disappointing for him to be told subsequent to his marriage that his wife in all probability would be childless, and I do not see how we can help him unless he stipulated as a condition of his marriage that his prospective wife should produce a certificate of normality. I do not think we have quite arrived at this stage of affairs at present, whether in the future we shall have help from legislation on this point I cannot say.

In another set of these fibroid cases decided symptoms are produced, and I consider that these women ought not to marry: the menorrhagia and dysmenorrhœa are both increased by married life, and if pregnancy takes place abortion is very liable to ensue. There are however a certain number of fibroid cases where pregnancy occurs and goes on to its normal termination, a living child being born. I have attended nine women in their confinements who had large uterine fibromyomata. One case I attended three times and one four times. All of these cases did well, and in one of them the result of repeated pregnancies was to cause total disappearance of the tumour of the uterus. The history was most instructive: at each pregnancy the uterine tumour increased in size; but during the involution of the uterus the tumour also underwent involution, reducing it considerably. Six months after the termination of the fourth pregnancy the tumour had entirely disappeared. Now, although this woman was cured of her uterine tumour by repeated pregnancies, I do not advocate this mode of treatment, and I should advise against marriage in any case of fibroid tumour of the uterus where the usual symptoms of that condition were present.

Where the fibroid tumour produces no symptoms and the contracting parties are made aware of the probability of a sterile life, then I see no reason to negative marriage. Each of these cases must be treated on its own merits, and we must bear in mind that the condition and position of the growth in the uterine wall may vary with time. I had under my care for some five years a patient with the growth in such a position as to cause profuse hemorrhage at the periods. By degrees this growth moved towards the peritoneal surface of the uterus, with the result of allowing the uterine mucosa to become healthy: the woman then conceived, and would have gone to her full time; but not knowing she was pregnant, I passed a sound into her uterus and produced an abortion. This case was very instructive, as the woman had regular monthly bleedings, no symptom of pregnancy, and a hard fibroid cervix; but she had two abdominal tumours at this visit, whereas six months before she had only one, and that one I had known of for years. Here the fibroid condition of the uterus masked the symptoms, and I suspected a second outgrowth from her uterus or possibly an ovarian cystoma. The mistake I made was, in not using the stethoscope over the new tumour; for if I had I should have heard the fœtal heart, and then possibly I should have done on purpose what I did by accident, as the fibroid growth interfered with the uterus maintaining its normal axis in the pelvis, and I have no doubt that the induction of an abortion was less risky than letting her go on to her full time.

There is no doubt that pregnancy complicated with a fibroid tumour of the uterus is a very much more risky affair than under ordinary circumstances. We have, in addition to the mechanical obstacle often offered by the fibroid, to deal with a phase in the life-history of these growths which is most important to bear in mind, and that is their tendency to necrose if their nutrition is interfered with.

This is a feature that all of us who have to treat these cases must always bear in mind. I have seen several women nearly die from septic changes occurring in the uterine tumour itself, due to its nutrition being interfered with in labour and sometimes due to direct injury in the confinement. I am sure the great point in managing a midwifery case complicated with a fibroid uterus is, not to let the uterus become tired out, for if you do you will be sure to have to face a bad attack of *post-partum* hemorrhage: we all know how prone a tired uterus is to bleed under normal circumstances, and it is infinitely more so when it is the seat of a fibroid tumour which interferes with the mechanism of complete contraction and retraction of the uterine wall; besides, in these fibroid cases you do not get the assistance from ergotin injections and the hot water douching, for the simple reason that the arrangement of the muscular fibres of the uterus is interfered with to a certain extent by the presence of the fibroid growth. Bearing this in mind, always have at hand perchloride of iron to produce a mechanical stasis, when you are no longer able to obtain the normal physiological process necessary for the arrest of hemorrhage.

The other evil of long continued uterine action is the direct interference with the nutrition of the fibroid growth, and that this is no imaginary danger I can prove by several cases of sloughing of the fibroids *post partum*, as well as the production of a low form of septicæmia which is exceedingly difficult to treat and may require removal of the whole uterus to save the life of the patient. In all the cases I attended I cut short the labour as much as possible, and in all the fourteen labours I did not have any complications. The early use of the forceps is indicated in these cases if the vertex present, and manual assistance at an early date in other presentations.

The question of malignant disease of the uterus rarely complicates my subject, as it is not common at the ordinary age of marriage, and when present would of course be recognised as a distinct bar to marriage. There are cases of malignant disease of the uterus where the main symptom of increased loss of blood at the catamenial periods has been put down to other causes, and women have married not knowing their condition and have become pregnant, this latter state being an exceedingly grave complication. Yet if the point I contend for be granted, these cases ought to be recognised and marriage interdicted. We all know how patients with cancer of the uterus go on for a long time without seeing a doctor, but then as a rule the women are either married or spinsters with no idea of marriage. The early stages and history of malignant disease are pretty well known to medical men and should be attended to, and any woman contemplating marriage with such suspicious symptoms must be examined with the view of ascertaining what her real state is; and if the condition is suspicious, then a given time should be allowed to elapse to enable the doctor to say positively what the disease is.

There are other pelvic conditions which I think should absolutely bar marriage, and one is such deformity of the pelvis that no living child can be born. Of course it is very easy to say that an abdominal section can be done and the child saved, but this mode of delivery is more serious to the mother than an ordinary labour, and if she cannot bring forth her child in the natural way she should stand aside and let a more fortunate sister have her chance. Most of the women I have seen with such a deformed pelvis have become pregnant without going through the formality of marriage, and so no one had the opportunity of advising against it. I do not think that in the better classes a woman with such deformity of the pelvis would think of getting married; and if she did, would most likely consult a doctor before she took such a step, and then the question of pregnancy must be faced.

I now come to consider very briefly the remaining cases. No woman ought to marry with an ovarian tumour or broad ligament cyst, as the dangers of pregnancy are no doubt greatly increased. I do not think we are likely to have to give an opinion on the marriage question here: of course, if we did we must say that, until the operation for the removal of the tumour has been performed and we know what the state of the pelvic organs is after the operation, we cannot advise for or against marriage.

Several women from whom I have removed one ovary for cystic disease have married and had children, and there has been no loss of sexual capacity. Again, in some of my cases in which I have removed both ovaries in married women there has been no loss of sexual power: this fact is important in deciding whether a woman from whom both ovaries have been removed is pretty much in the same position as a woman who has ceased menstruating at the ordinary time of life. The very few cases I have seen have certainly led me to give the opinion that the only difference will be that there will be "no family," and that otherwise there is no reason to negative marriage. The various forms of tubal disease most frequently cause alterations in the menstrual life, producing pain and excessive hemorrhage, and if my advice is acted on the pelvic examination will reveal the disease and marriage must be vetoed.

There are many conditions which interfere with the due performance of the marriage contract, but most of them are brought to our notice after marriage and can be remedied. The great point is, to prevent women marrying in ignorance of such a condition as cannot be remedied but only made worse by marriage.

I have purposely limited this address to the evils of women marrying with pelvic abnormalities. I cannot this evening go into a subject that I have given much attention to, viz., the influence of pregnancy in chronic and acute diseases. There is no doubt but that there are several chronic diseases that ought to act as a bar to marriage, some on account of the woman herself and some on account of her offspring, but this subject I cannot discuss now; nor can I open up the important subject of the effect of pregnancy in acute diseases, a subject of great importance and one that I had the opportunity of working at in my earlier years of practice when I did a good deal of general work.

My object to-night is to bring before you what I consider to be a very great evil, and ask you to help to limit it as much as possible. I must apologise for incorporating such a poetical subject as marriage into the dull prose of a medical address.

